# Challenges in accessing patient-centered care and patient empowerment in selected Ghanaian hospitals

**DOI:** 10.4102/hsag.v29i0.2623

**Published:** 2024-11-26

**Authors:** Ruby V. Kodom, Robert T. Netangaheni

**Affiliations:** 1 Department of Health Studies, College of Human Sciences, University of South Africa, Pretoria, South Africa; 2 Department of Health Services Management/DE, School of Continuing and Distance Education, University of Ghana, Accra, Ghana

**Keywords:** patient-centred care, patient empowerment, resource availability, financing, communication, transportation, skilled workers, waiting times

## Abstract

**Background:**

Patient-centred care (PCC) and patient empowerment (PE) are crucial for better healthcare outcomes, in lower-middle-income countries like Ghana, which continues to encounter many challenges.

**Aim:**

The study sought to determine the factors affecting the implementation of PCC and PE in Ghana through the voices of patients and healthcare providers.

**Setting:**

The study is based in Ghana, West Africa, and includes three healthcare facilities representing primary, secondary and tertiary care.

**Methods:**

A qualitative exploratory descriptive research design was employed to investigate the study’s objective by engaging healthcare workers and patients in selected facilities through purposive sampling. While 33 healthcare service providers participated in in-depth interviews, focus group discussions were held with four patient groups. The collected data were analysed thematically to identify key themes and insights.

**Results:**

The analysis revealed three overarching themes: organisational-, individual-, and environmental-level factors influencing PCC and PE. Findings presented under 10 sub-themes show that resource constraints and staff shortages hinder PCC, while patient agency and communication impact PE. In addition, the ability to pay and geographical barriers further hinder access to patient-centred services, affecting overall healthcare delivery.

**Conclusion:**

The findings from this study emphasise that without system-wide interventions to address these issues – including improving resource allocation, enhancing communication, and reducing geographical and financial barriers – achieving Universal Health Coverage (UHC) by 2030 remains highly aspirational.

**Contribution:**

The contribution of the study is inherent in the relevance of contextual findings towards improving health service delivery.

## Introduction

Patient-centred care (PCC) has been recognised globally as essential to improving healthcare outcomes. Patient-centred care shifts the focus from the provider to the patient by prioritising patient needs, values and preferences during healthcare decision-making. This concept goes beyond clinical interactions to include emotional, psychological and social factors that affect patient well-being. Meanwhile, patient empowerment (PE) is a process by which individuals gain greater control over decisions affecting their health (Pekonen et al. 2020). This concept hinges on ensuring that patients are well-informed, actively engaged and able to make choices about their care. Empowered patients are better equipped to manage their health conditions, particularly in non-communicable diseases (NCDs) where long-term self-management is essential.

There has been a concerted effort to strengthen healthcare systems. The World Health Organization (WHO 2018), with a focus on making healthcare more accessible, equitable and patient-centred, leveraged the principles of primary healthcare for actualising Universal Health Coverage (UHC) aspirations.

Regionally, African countries, including Ghana, have been working to align their health systems with the global health goals; however, they face unique challenges such as resource limitations and workforce shortages.

Ghana has since independence, displayed a commitment to providing healthcare that is of good quality, for its citizens through several health reforms (Nkrumah & Abekah-Nkrumah 2019). Despite many recent efforts such as the National Healthcare Quality Strategy (NHQS) to address quality and patient satisfaction gaps, the reality remains that care delivery is often more provider-centred, with healthcare workers overwhelmed by workloads, leading to reduced time for meaningful patient engagement (Adu et al. 2022). Challenges to PCC include health worker shortages, long waiting times and systemic inefficiencies in healthcare systems, all of which can erode patient trust and satisfaction (Sidani & Fox 2020).

In Ghana, studies have highlighted issues such as insufficient staff-patient ratios, inadequate communication and inconsistent delivery of patient education, all of which detract from the implementation of PCC (Nkrumah & Abekah-Nkrumah 2019). Concerning workload, Ghana has implemented policies that have expanded the pool of local training institutions (Ministry of Health [MOH] 2014; WHO 2020). The MOH in Ghana has invested in revamping the human resources base through the completion of the ‘human resources for health’ policy in 2020 (MOH 2021) and the recruitment of over 52 000 health workers in the same year 2020 (MOH 2021). A review of Ghana’s health human resource stock shows significant improvement in the doctor-to-patient and nurse-to-patient ratios (MOH 2021). The MOH reports improvements in the distribution of trained midwives across the country. However, the increases in health workforce production are inadequate and unfairly distributed (Acquah-Hagan et al. 2021) (Korah, Nunbogu & Ahmed 2023). While rural populations remain deprived of speciality services (Peprah et al. 2020; WHO 2020), urban areas deal with inefficiencies in the distribution and scheduling of workers.

Barriers to PE in Ghana include poor health literacy, inconsistent communication between providers and patients (Maame et al. 2019), and limited access to health information (Amoah & Phillips 2018; Amoah, Nyamekye & Owusu-Addo 2021; Wu et al. 2021). These factors are particularly pronounced in areas, where resource constraints and cultural beliefs about health can further disempower patients (Peprah et al. 2020). For quality care to be prioritised, supporting systems would have to be implemented. Furthermore, while policies such as Ghana’s Patient Charter aim to protect patient rights and promote empowerment, many patients remain unaware of these protections, limiting their ability to advocate for themselves (Nkrumah & Abekah-Nkrumah 2019). Healthcare service delivery takes place within organisations and thus healthcare organisations are central to the strengthening of health systems (Swanson et al. 2012). These organisations require the right environment and resources to perform their mandate for patient satisfaction. This environment is one that is welcoming, clean and responsive to the needs of patients and staff, among others.

Patient-centred care and PE remain critical for improving healthcare outcomes in Ghana. However, structural, cultural and resource-related challenges continue to impede the full realisation of the health and well-being outcomes that these concepts produce when integrated into systems of healthcare delivery. This study seeks to explore these challenges further, identifying and exploring the antecedent causes with a view to make them areas of focus for improvement of healthcare interventions.

## Problem statement

Universal Health Coverage aims to ensure that all individuals have access to quality health services without financial hardship. Despite significant investments in health systems and financial models to enhance healthcare accessibility, there remains a significant gap in achieving effective UHC, particularly in many African countries, including Ghana (Olu et al. 2019; WHO 2020). The global health community recognises that while financing is crucial, it is insufficient on its own to ensure quality healthcare (WHO 2020).

Ghana has made structural adjustments and investments in health, yet progress towards UHC remains inadequate (Lozano et al. 2020). The country continues to face challenges in healthcare delivery, including insufficient health worker-to-patient ratios and inadequate healthcare quality. As reported in the MOH’s medium-term development plan (2014–2017), the doctor-to-patient ratio was 0.10 per 1000 population, and the nurse-to-patient ratio was 1.14 per 1000 population, both below the WHO-recommended threshold of 3.4 skilled health workers per 1000 population (Amoah et al. 2021; MOH 2014, 2020; WHO 2018).

Failure to address these challenges can lead to worsening health outcomes, increased mortality because of poor care, and persistent inequities in health service delivery (Kruk & Pate 2020). The persistence of long waiting times, short consultation periods and unauthorised payments undermine patient trust and satisfaction, further hampering the effectiveness of healthcare delivery (Nkrumah & Abekah-Nkrumah 2019).

This study aims to explore the local context of healthcare delivery in Ghana, focussing on the factors that influence PCC and PE. By identifying the real contributors to healthcare successes and shortfalls, the study provides insights that can inform targeted interventions to improve PCC. Addressing the reductionist, one-size-fits-all approaches, this research seeks to enhance the understanding of how to achieve a more patient-centred, effective health system in Ghana.

### Study purpose

The study sought to identify the factors that challenge the actualisation of PCC and PE.

To do this, the following research questions were posed:

- *What organisational level factors affect the extent to which PCC and PE is implemented?*
- *What systems and standardised processes exist for stakeholders to be guided by?*
- *What are the individual-level conditions that determine whether patients receive PCC or PE?*
- *What environmental factors contribute to the presence and/or absence of PCC and PE?*

## Research methods and design

The study adopted a qualitative exploratory descriptive design to investigate the factors that influence the implementation of PCC and PE in healthcare settings. This design is appropriate for exploring complex, multifaceted issues within healthcare systems, as it allows for the collection of detailed data to identify patterns, insights and challenges (Creswell & David Creswell 2018). Rather than focussing on solely individual experiences, the study aimed to explore systemic and organisational-level factors, drawing on the perspectives of both healthcare workers and patients. This method facilitated a deeper understanding of the barriers and enablers to delivering PCC in Ghana’s healthcare facilities, particularly in the context of achieving UHC.

Descriptive research designs aim to observe, describe and record responses from patients, increasing the researcher’s understanding of the challenges influencing the delivery of PCC and PE in Ghana.

### Setting

The study was conducted in the capital of Ghana within the Greater Accra Region. The region was selected as it provided a good mix of facilities at the different levels of care. It was also of interest because, according to the Ghana Statistical Service, it is one of the smallest regions in Ghana by land area, with the second largest population of about 5 455 692 as of 2021.

### Sample and sampling method

A sample of three hospitals within the region took part in the study and served as data sources for healthcare at the primary, secondary and tertiary levels of care. The hospitals were sampled judgementally out of a total of 111 hospitals and 32 health centres, to ensure a representation of the three-tier service structure that is used in Ghana. Non-probability sampling methods specifically, purposive and convenience sampling were used to identify and recruit study participants. Health workers (nurses, doctors, and administrative staff) were sampled purposively specifically based on their expertise as care providers from the various service departments. Patients or clients were sampled according to their convenience and availability. The data from these two population groups were key to presenting a nuanced view of the study phenomenon, in a way representative of the major stakeholder views. A total of 33 health workers, sampled from the Korle Bu Teaching Hospital, Greater Accra Regional Hospital and the Frafraha Health Centre, took part in the study. An additional 28 patient/client participants were part of the four focus groups that were carried out across the three health facilities. Each focus group had seven patients participating. The criteria for inclusion was that participants had to be patients who received care at the respective facilities.

### Data collection and tools

Data were collected using interview techniques from the natural setting of the study sites and participants. Driven by the need to know how PCC is operationalised in the context of a developing country (Mabbott 2013), the authors employed in-depth interviews to understand how healthcare workers execute their mandate in the interest of population health and well-being. Data were collected from October 2022 to July 2023. Individual semi-structured interviews were conducted with the health workers to understand the factors influencing PCC, PE, and ultimately, UHC. Some examples of the questions asked are ‘Based on your experience, what are some of the things that influence PCC?’ or ‘How can we empower patients to support the healthcare system?’ A pilot was employed to confirm the validity of the questions and lessons from that initial study were used to tweak the interview guide so that there were two interview guides. One for healthcare providers and one for patients. Follow-up interviews were conducted to gather additional information from the health workers and to authenticate data. Interviews were conducted with the participants who consented and lasted on average about 50 min. Interviews were audio-recorded with the permission of the study participants. Notes were taken during interviews. The services of research assistants were enlisted to facilitate the interview process and data transcription. A translator was engaged where necessary. The distress protocol in place made use of the services of a psychologist to help manage unpleasant situations that had to be recalled as part of the data collection process. None of the participants required the services of this doctor.

To garner the patient perspective, focus group discussions (FGDs) were conducted with patient participants given that they are recognised as a standard way of assessing patient satisfaction with healthcare services received. The focus groups included seven males or seven females in each session. The decision to separate the different groups was taken to reduce the likelihood of bias and dominance of one gender. These interviews elicited responses, which were rich descriptions to answer the study questions.

### Data analysis

Qualitative data analysis aims to derive meaning from the participants’ spoken words, forming the foundation of the data analysis process. Miles and Saldana (2014) emphasised that the data analysis should commence with the data collection process. Participant responses prompted probes and follow-up questions, allowing deeper insights. Close listening and note-taking during and after interviews helped identify meaningful connections and develop initial codes (Williamson, Given & Scifleet 2018). These codes were then organised using inductive reasoning and were clustered based on commonalities to reveal themes. Initially generated themes were reviewed within the context of the study’s objectives and research questions. Subsequently, the reviewed themes were defined, and the presented findings were guided by the developed themes. The thematic analysis of data follows the steps outlined by (Braun & Clarke 2006), offering flexibility in approaching data and allowing for the assessment of the research participants’ different perspectives. Moreover, it aids in understanding areas of consensus and disparities among participants. This approach to analysis bolstered the study’s rigour by necessitating adherence to a structured process.

### Trustworthiness

Trustworthiness is the measure by which the findings of a qualitative study can be trusted based on the extent of rigour that is inherent (Nieswiadomy & Bailey 2018) thus pointing to quality research. The criteria for setting the trustworthiness of the study following Guba’s model are credibility, transferability, dependability and confirmability,

The credibility of the research is established by accurately recording, systematising and fully disclosing the methods used to arrive at the data findings. Credibility was achieved as the researchers were able to obtain a clear fit between the views of the respondents and how they have been presented through prolonged engagement by doing follow-up visits after the initial data collection process, participant checking and triangulation using multiple data sources. Peer examination was followed to ensure that the researchers biases did not affect the study outcome.

Transferability, which is one of the four components of Guba’s model of trustworthiness, seeks to show how the results of the study can be reproduced given similar contexts and conditions. Gray and Grove (2017) state that transferability is supported when a researcher gives a detailed description of their study context. Data saturation regarded as an enhancement factor of transferability (Denzin & Lincoln 2018) was used to determine the study sample size. This was achieved by continuously collecting data until no new themes or insights emerged.

According to Flick (2018) dependability is concerned with the ‘documentation of steps taken, and decisions made during analysis’. Dependability was ensured by taking field notes and observing non-verbal cues throughout the interview process.

If the data collected can confirm that the results and conclusions of a study are true, then confirmability is attained (Nowell et al. 2017). Confirmability was ensured by triangulation, specifically in relation to the population groups recruited and the methods used to collect data. Bracketing was employed to engage both healthcare workers and patients, helping ensure that the findings authentically reflected their experiences and views. Quotation marks preserved the richness of the data by conveying participants’ views.

### Ethical considerations

The study received ethical approval from the University of South Africa’s College of Human Sciences (reference number 69969213 CREC_CHS_2021), as well as from the ethics review committee of the Ghana Health Service and Korle Bu Teaching Hospital. Written permission was granted by the hospital management, and participants provided informed consent after reviewing the information sheet. Efforts were made to ensure the anonymity and confidentiality of participants. Participation was entirely voluntary, and participants had the right to withdraw their consent at any stage of the data collection process.

## Results

### Sociodemographic characteristics

A total of 28 patients or clients took part in this study. The majority of the patient participants were between 20 and 39 years of age. There was an even split of males and females. For the healthcare worker population group, the sample comprised of 18 nurses, 12 doctors and 3 administrators. There were 10 males and 23 females. Most of the healthcare provider participants were aged between 30 and 39 years.

### Themes

The data analysis produced a total of 3 main themes and 10 sub-themes (Table 1).

**TABLE 1:** A summary of themes and sub-themes.

Themes	Sub-themes
1. Organisational level factors affecting access to PCC and PE	1.1 Resource allocation and its impact on patient-centred care and patient empowerment 1.2 Health workers’ well-being and burnout 1.3 Healthcare service structure and accessibility
2. Individual-level determinants of PCC and PE	2.1 Financial constraints and the role of health insurance in healthcare accessibility 2.2 Extended waiting times as a deterrent to seeking and receiving timely care 2.3 Effectiveness of patient-provider communication in fostering PE 2.4 Influence of attitudes on patient experience and engagement
3. Environmental-level factors affecting PCC and PE	3.1 Patient safety and security as prerequisites for PCC and PE 3.2 Facility cleanliness and management practices 3.3 Geographic accessibility and transportation barriers to PCC and PE

PCC, patient-centred care; PE, patient empowerment.

With the conversation on PCC and by extension PE in healthcare featuring heavily on the agenda of health leaders in lower-middle-income countries (Anabila, Anome & Kwadjo Kumi 2020), the study’s findings are classified under three levels to cater for the multidimensional nature of quality. The results presented are based on participants’ experiences as caregivers or patients, and juxtaposed with the literature review. The theme of systemic or organisation-level factors affecting access to PCC and PE is broken down into three sub-themes. This classification allowed for more in-depth detail of the issues presented.

#### Theme 1: Organisational-level factors affecting access to patient-centred care and patient empowerment

The factors that were found to affect PCC and PE at the organisational level included: service structure, staffing and the availability of resources. The organisation of healthcare in Ghana adopts a top-down approach to management (Appiah-Agyekum 2020). For PCC and PE to be actualised, the management of healthcare organisations need to commit by providing the vision, direction and integration of strategy for PCC values into day-to-day facility service output. Key participants shed light on some organisational factors that impact their ability to give care that reflects patient-centredness. These factors included availability of resources, staffing and service structure and availability.

**Sub-theme 1.1: Resource allocation and its impact on patient-centred care and patient empowerment:** Participants in the study demonstrated an appreciation for the difference that having adequate facilities, consumables and equipment makes in the delivery of care to patients. Resources that were not available that affected PCC included consumables such as syringes and paper towels. In addition, participants highlighted how medicines and medical equipment such as wheelchairs and machinery affected PCC. The experiences of health worker participants with how resource availability influences the type of care received are highlighted by the following quotes, participants were identified using codes, which were participant number, facility name and type of interview (Respondent [R], In-depth Interview [IDI], Facility KBTH, Greater Accra Regional Hospital [GARH], Frafraha Health Centre [FHC], focus group discussion [FDG]):

‘At times lack of resources like gloves, and syringes. Nowadays, Hospital T, we have BCG, but we don’t have a syringe.’ (R2, IDI, KBTH)

The issue of medical equipment also affected PCC:

‘Hospital equipment is not enough so maybe someone may need five minutes or three minutes with a particular machine and because there is too much pressure, you reduce to two or one minute and move to the other person.’ (R9, IDI, GARH)

The issue of maintenance culture or the lack thereof was brought up in the instance where service implements did not receive the needed maintenance:

‘And then also, maintenance culture. Because if am supposed to be using a BP machine or for some time it’s developing a fault and there is no prompt intervention of replacing it, at the end of the day, you will be giving the patient wrong figures.’ (R5, IDI, KBTH)

The healthcare workers also described the lack of infrastructure, which affected delivery of healthcare services in private settings. The problem of inadequate space negatively affects patient-centredness in the sense that patients’ dignity and sometimes privacy are sacrificed to structural constraints.

For instance, in referring to consultation in the quote that follows, a health worker had this to say:

‘For us, we do it openly where we are. So sometimes counselling somebody, the client sitting around you, behind you can listen to what you are saying.’ (R5, IDI, KBTH)

Healthcare participants described the organisational-level factors that affected the provision of PCC and PE from the perspective of available resources. These factors included equipment, infrastructure and medical consumables, which are necessary to ensure that patients always receive quality healthcare.

**Sub-theme 1.2: Health workers’ well-being and burnout:** The overworked healthcare professionals impacted the quality of care and patient trust. This theme captures how these factors influence patients’ experiences and access to quality healthcare, ultimately affecting their empowerment and overall satisfaction with the healthcare system in Ghana. Staffing is essential to the goal of UHC, and health workers are identified as the basis on which quality services thrive. Participants, from the various backgrounds sampled, stated that staffing significantly affected the achievement of PCC in Ghana. Staffing is also identified as one of the key dimensions of Patient Safety Culture within healthcare organisations This implies that to ensure quality there should be skilled workers in healthcare facilities. The quotes from the participants showing how inadequate staffing affected PCC are as follows:

‘Staffing is a problem. So, we are severely understaffed, we are overstretched. I mean that has its implications because then for the staff, you end up frustrated because you are not able to give your best. You end up making mistakes because again if you are seeing a large number of patients, the tendency for things to fall through the cracks becomes higher.’ (R15, IDI, GARH)

Another health worker stated:

‘You could have a well-structured facility with all the logistics and what have you but if your staff strength is not enough, we can’t give you that quality care that you need.’ (R11, IDI, GARH)

Adequate human resources are synchronous with quality care delivery which ensures the well-being of patients and staff. The current situation breeds burnout among health workers. Nurses have the highest vulnerability to burnout among the various health worker groups in Ghana. This argument is supported by the following quote:

‘Human resources affect the quality of care because, in this particular facility, we don’t have a records officer, so it is the nurses we use. We don’t have a NHIA officer so it is the nurses we use. We don’t have an accountant. It is the nurses we use. So, in every place here we use our nurses who are not qualified to do the work so, there are certain times there are some lapses and you may not know, when you ask them, they’ll say I am not a record officer. I’m just trained on the job to do it.’ (R7, IDI, FHC)

Some health worker participants confessed to having developed chronic medical conditions because of the persistent high workload:

‘for instance, at my side, I’m the only nurse manning room 5,6,7,8 and 9, five rooms. I move from here to there to there, and this one is waiting for you, this one wants you to do this. I mean, I’m killing myself. I have recently been diagnosed with hypertension at my age.’ (R8, IDI, GARH)

Despite the MOH’s efforts to train and recruit more health workers, Ghanaian hospitals and healthcare centres continue to suffer from a shortage of adequate staff.

**Sub-theme 1.3: Healthcare service structure and accessibility:** Providing services that are reflective of the needs of the people contributes to the utility of healthcare services and can influence the health-seeking behaviour of communities. The healthcare workers described how some services such as laboratory services are not available, which would then require referral. The quotes that support this are as follows:

‘In this community, they call here what, “Adenta Korle Bu”. They have a name they call this place because this is the only government facility around. They say when you come here you won’t get anything. That is what they say … because sometimes they come here on weekends and the lab is not working.’ (R7, IDI, FHC)

Furthermore, the services available at a facility influence PCC and PE by ensuring timely access to needed services. The following quote supports this point:

‘Because here it is a primary care facility, and there are only midwives who work here. We don’t have a theatre, so there is no doctor. So, whatever is above the midwife, the midwife has to refer, it has to be so early to avoid complications.’ (R6, IDI, FHC)

Study participants sampled from the tertiary hospital however indicated that patients accessed all necessary health care services with ease and this enabled PCC. The following quote shows how service provision was accessed at a tertiary facility:

‘I think the existence of the facility makes it easier for people to access help because this, as we know already, is a referral centre. We see more complicated cases and because the facility is there, when it is discovered that someone has some complex case, is easy to refer the person to the facility for care.’ (R13, IDI, KBTH)

The responses indicate a need for increased sensitisation on the levels of care and service categories that are assigned as per the three-tier referral system of the Ghana Health Service (GHS). This is important to address the perception that some healthcare facilities do not have adequate services and rely on the referral system for effective medical care. The responses also suggest that the facilities sampled are functioning to meet the needs of community members with varied results and levels of satisfaction.

#### Theme 2: Individual-level determinants of patient-centred care and patient empowerment

This theme focusses on identifying and understanding the various individual-level factors that impact the delivery and perception of PCC. It investigates the role of empathy in empowering patients highlighting the role of the approach and behaviour of health professionals towards patients. It explores how trust and rapport contribute to PCC and what happens when it is lacking. Some challenges in effective communication between patients and healthcare providers are presented here. It also examines how extended wait times affect patient experiences and expectations of the healthcare system.

**Sub-theme 2.1: Financial constraints and the role of health insurance in healthcare accessibility:** In Ghana, financing for health is performed through a social financial protection scheme, the National Health Insurance Scheme (NHIS). Despite the presence of the NHIS, participants described challenges in affording healthcare. The responses of the patients are shown as follows:

‘It covers some of the labs because when you don’t have the insurance, the bill is higher.’ (R4, FGD, FHC)

‘I have it but it’s not working here … the bills are high. It doesn’t cover anything … nothing, when you see my bills right now, you’ll, marvel … It has to cover, some, parts of our bills so that we get relief because we are suffering, Ghana is very hard. Now they’ve discharged me, I don’t have money to pay my bills and I’m here. I can’t run away too … the medicine that I bought, 90 million [*GH 9000*] but then my bills … GH 33 000. Why? Whilst I’m having NHIS.’ (R5, FDG, GARH)

Based on these reports by the patients, the NHIS’s effects on reducing healthcare costs were minimal. Healthcare workers also corroborated the patients’ findings regarding the effectiveness of the NHIS:

‘This insurance, I don’t know how it’s helping the patients because aside from the consultation fee they are paying, you will be on insurance and after discharge from the ward, the bills they pay.’ (R4, IDI, GARH)

‘So, you are coming with NHIS but it’s just covering your folder. So, you must buy your medication, lab, you will pay … most at times it covers just paracetamol or something, while the remaining expensive drugs are not covered.’ (R3, IDI, FHC)

**Sub-theme 2.2: Extended waiting times as a deterrent to seeking and receiving timely care:** Timeliness is one of the hallmarks of quality healthcare provision. The Institute of Medicine (IOM) prescribes a 30-min wait time for 90% of patients within the time of their appointment. Patient or client participants in this study shared similar sentiments on this as reported in the following response:

‘You come early, and you just sit and waste your productive time. Then the earliest you can be taken care of is after spending over 4 hours. Less than that, me, I’ve not experienced less than that hour here before … I’ve been coming to Korle Bu since 2009.’ (R5, FGD, KBTH)

‘When I’m coming to Korle-Bu, I take it as if today, I’m going to my hometown. Yes, I’m going to my hometown, I’m not coming back. I will sleep there so tomorrow morning; I will be back. So, when I’m coming to Korle Bu, I tell you, I have everything in my bag.’ (R2, FGD, KBTH)

The healthcare workers were aware of the long waiting times and how it affected the quality-of-care provision. This was evidenced by their responses in the following quotes:

‘One of the things that influences the quality of care, is time. Our patients normally complain of the time they spend at the hospital.’ (R4, IDI, GARH)

‘If I should come and I have to come in like 6 o’clock and I have to wait till sometimes I have to see the doctor by 2 or 3 o’clock[*pm*] then with that, next time I wouldn’t want to come.’ (R5, IDI, KBTH)

Although patients at the larger hospitals reported long waiting times to receive services, some patients who were sampled from the health centre stated that they received services promptly. The quote to support this is as follows:

‘Here I realised that within an hour or two, you get cared, good care and you can go off to do the other things you have to do. To me, time-wasting here is minimal … they have other facilities that they can also help with care. You don’t have to go around looking for, especially when it has to do with vaccinations.’ (R7, FGD, FHC)

Noteworthy, at the same healthcare facility, the healthcare workers highlighted that they were conscious of waiting times and strived to serve patients timeously.

**Sub-theme 2.3: Effectiveness of patient-provider communication in fostering patient-centred care and patient empowerment:** Good communication contributes to a positive patient experience whereas quality is lost when communication is compromised because of language differences. Patients cannot be empowered without the sharing of adequate information. This implies that without good patient-provider interaction, the quality of engagement towards achieving positive health outcomes may not be realised. The patients reported negative experiences regarding how some healthcare workers speak to them. These experiences included being shouted at and not being provided with adequate information. The quotes to support this are as follows:

‘Like menstrual cycle problems. I used to face a whole lot within there. So, anytime it comes, I have to go to the hospital. When I go to the hospital, my family knows what is wrong with me. Like when I go, they talk to me rudely, ‘you’ve gone to do abortion and you’re here’ screaming’ [*tuts*] it’s like, ah! you don’t know me and what is wrong with me. You have to ask me … but here you are like abusing me.’ (R1, FGD, FHC)

A challenge with communication as captured here shows the need for patients to receive adequate explanation from care givers:

‘With the lab request, you have not told me what the issue is. You give me a list to go for drugs maybe if I have malaria, they don’t say it … After, he told me Take, go for drugs. I’m done with you. Hmm “You are done with me” I asked, Doctor, what’s wrong with me? I’ve done the labs, what’s wrong with me, tell me. He didn’t tell me.’ (R3, FGD, FHC)

Patient participants are dissuaded from coming to the healthcare engagement as equal partners. This response shows the prevalence of the paternalistic approach to service delivery, which frowns upon questions being asked by patients.

This response from a health worker participant should be the appropriate scenario for what was experienced in the previous quote:

‘I feel the patient should be heard clearly. The patient should be made to understand what is wrong with him [*or*] her. If it is a procedure, you need to let the patient understand what procedure it is and what you are doing.’ (R8, IDI, GARH)

The healthcare workers also accept that there are communication challenges and language barriers when interacting not only with patients but among themselves also. A health worker participant observed the following:

‘I think we have to organise a workshop for staff on their communication skills because at times even when you meet a staff and the way the staff will be talking with you, it can even kill your soul more … we have to train our staff how to communicate with the patients.’ (R2, IDI, KBTH)

Communication is the bedrock of effective PCC and PE Good two-way communication enables patients to express themselves and for health workers to build trust and gain deeper insight into patients. Ghana is a multilingual country with over 50 different languages. Majority of the people speak at least one of the major languages and the official language is English. In a referral hospital typically, communication can be tough when patients and providers cannot understand one another.

In expressing concern for the communication barriers stemming from differences in language, one health worker participant said:

‘I’d add language barrier. The person would like to express himself or herself in a way that you might understand but we have what we call the language barrier the person doesn’t understand what you are asking, and the person will be giving a wrong answer to what the issue is. You will be limited because you are not getting each other.’ (R2, IDI, FHC)

Thus, language barrier was mentioned as a challenge in the delivery of quality healthcare.

**Sub-theme 2.4: Influence of attitudes on patients’ experience and engagement:** Another area of influence that emerged as a facilitator and or barrier to PCC is the attitude of health workers as well as the attitude of patients to the care interaction process. One health worker described how their attitude was not influenced by educational levels as follows:

‘Being educated and being qualified doesn’t affect your attitude in any way … People’s attitudes form, as a matter of their relations, their experiences in the past, the environment they grow in, and how their parents groom them. So, your education naturally, I mean from my point of view, has nothing to do with how you conduct yourself.’ (R8, IDI, GARH)

According to some patient participants in the focus groups, some health workers (doctors and nurses) showed a negative attitude by appearing unconcerned and were not empathetic to patients who were in pain or too weak to care for themselves or were too distracted to care. The quotes to support this are as follows:

‘And those [*nurses*] who do that particular thing are the ones who have never given birth before, that is, excuse my language … because if you have been through this before I don’t think you will see your fellow suffering or going through such pain and then you talk to him or her anyhow.’ (R2, FGD, GARH)

‘Imagine a pregnant woman comes, she’s suffering. That is when they will be on their phones laughing [*or*] giggling. When you get angry and comment, they tag you as this patient is not good … the person is in pain, you have to care for her, they will be on Facebook and be doing this, they will be laughing.’ (R3, FGD, FHC)

‘They act as if you are bothering them. The job they are doing, it’s, it’s like a burden. No love, no love.’ (R5, FGD, KBTH)

However, some health worker participants were aware of the impact negative attitudes as evidenced by the following quote:

‘If the patient had should come here and you don’t interact with that patient very well, bet me the patient will be psychologically traumatized.’ (R5, IDI, KBTH)

Health workers were also of the opinion that some patients are ‘difficult’. This labelling can compromise the type of care such patients may receive:

‘We normally get stubborn patients … because at times a doctor will prescribe, maybe write a request for the patient to come and check, and the patients will come and sit there ‘ah, this thing if I say I won’t do it what will happen?’ (R2, IDI, KBTH)

Health workers recognise the need for patients to engage with the healthcare system with an openness to achieve the shared outcome of patient health and well-being:

‘The cooperation and the willingness on the part of the client, yes, will help us to reach our target … Also, the treatment will be successful, not because we are giving any outstanding care.’ (R8, IDI, FHC)

The way health workers behave towards clients is important for building trust. Therapeutic communication goes a long way to enhance the physical and psychological health of patients.

#### Theme 3: Environmental-level factors affecting patient-centred care and patient empowerment

Healthcare service delivery is carried out in an environment that is influenced by physical, social and policy structures. While being satisfied generally with health worker interactions, patients primarily expressed concern about finding their way around hospitals and accessing services such as pharmacies, laboratories and other diagnostic services. The quality of the work environment influences the quality of care received as shown by the following quotes:

‘You know, we have physically challenged people. It is hard to get up the stairs, then there is no way if someone does not have a son or someone in your family who can carry you, then.’ (R4, FGD, KBTH)

The healthcare workers were in agreement with the patients that the physical environment of the hospital also plays a critical role in ensuring PCC and improved health outcomes. One health worker stated:

‘The environment also plays an optimum effect on patient recovery. So, it depends on the environment, then staff interaction with the patient and then the quality of the services that they kind of like access in the facility as well.’ (R5, IDI, KBTH)

**Sub-theme 3.1: Patient safety and security as prerequisites for patient-centred care and patient empowerment:** The physical environment, from the viewpoint of security, also affects patient health outcomes and PCC. Healthcare providers emphasised that work conditions should benefit both patients and staff. For instance, a participant described how access is denied to patients at night because of a lack of security for health workers during the night shift:

‘Most of the time, in the night, we don’t have a straightforward security service working here. And then opposite us is a pub or a bar and they smoke, and you see, we are all females. In the night, it’s two nurses here. And because they are women, when they come and it’s like 10 pm, they lock up. So, when you come here in the night as a sick patient, you will assume we are not working unless you knock for them to open.’ (R3, IDI, FHC)

Concerns were raised about patient safety in terms of ensuring confidentiality of problems discussed. The absence of adequate facilities were identified as a source of constraint on service delivery. The following quote from a healthcare provider participant captures this:

‘If you are saying focus antenatal care, then you need some kind of privacy, but then when the patient is here and then maybe if the patient is talking out like me, somebody will hear. so, let’s say inadequate facility.’ (R3, IDI, GARH)

Without adequate facilities that are always secure, safe spaces cannot be ensured for patient-centred interactions to take place.

**Sub-theme 3.2: Facility cleanliness and management practices:** The participants perceived that facilities for healthcare services were inadequate and did not meet general standards of hygiene. The inadequate cleanliness levels posed a risk of nosocomial infections for patients and health workers. In their descriptions, participants highlighted that there was a culture of lack of maintenance that seems to permeate most infrastructure in the country. Patients and health workers avoided using the washrooms in the hospitals. A health worker stated:

‘If the washrooms are not in good standing, they are not cleaned … One thing is that even if you keep urine for a long time, it also has health implications. So why then do I have to keep the urine? … once it is going to affect my health, and am keeping it because the washroom is not good, then the next time I shouldn’t even come to that facility because it will have added to the problem that you are already having.’ (R5, IDI, KBTH)

The patients also shared a similar view regarding the cleanliness of the washrooms. The following quotes summarises how patients view these facilities:

‘The washroom is very bad. If you are not careful, you will pick up infection.’ (R5, FGD, KBTH)

‘[*D*]on’t know if it’s me that is, you know, find things offensive. Using the washroom, I don’t pee. It’s like if I have to pee and you see me there … unless it’s an emergency or something. And I wonder, do they also go there, or do they have a special place.’ (R4, FGD, KBTH)

The lack of adequate cleanliness requires urgent attention as it counteracts the goal of providing universal access to quality healthcare services.

**Sub-theme 3.3: Geographic accessibility and transportation barriers to patient-centred care and patient empowerment:** The participants also described environmental factors that affect PCC – access including geographic access challenges. The distances people must cover to receive medical attention, and the means and cost of transportation were identified as environmental factors affecting healthcare access. Travel time is important when medical emergencies occur. In this study, healthcare workers and patients spend a considerable amount of time getting from their homes to the facility where they work or receive medical care. This contributes to worker and/or patient fatigue and can affect the therapeutic relationship required for effective PCC and PE. The responses from the healthcare workers are shown as follows:

‘One of the challenges is the road network, we have a very deplorable road network. And most clients are also living a bit far away which makes it difficult. When they need to come, sometimes the road prevents them.’ (R4, IDI, FHC)

‘Getting here is not easy at all. That one is the client’s challenge, me too my challenge.’ (R6, IDI, FHC)

On the other hand, some hospitals are accessible and participants shared their experiences of accessing their workplaces:

‘It is easy to get transportation in and out, so people come, yeah, people come from far … it’s just the fact that it’s in the centre of town. Getting transportation in and out is easy.’ (R13, IDI, GARH)

## Discussion

Universal Health Coverage encompasses the provision of healthcare at a cost that the population can afford (Novignon, Lanko & Arthur 2021). In Ghana, trained health workers play a vital role in providing PCC and empowering patients to ensure safe and quality healthcare. Many studies (Ahmat et al. 2021; MOH 2021; Scheffler et al. 2018) agree that achieving the goal of UHC depends on the availability of a robust health workforce. Health workers identified adequate human resources as a key contributing factor to PCC and PE at the organisational level to counteract. health worker burnout, which is characterised by an intense feeling of exhaustion, low self-esteem and poor job performance (Odonkor & Frimpong 2020). The trend of increasing the health of workforce indicates progress in achieving the goals of the WHO (2020) global strategy on human resources for health (Nyoni et al. 2022). Considering these reported strides made towards addressing the existing healthcare human resources, the study findings contradict the findings of earlier studies (Boniol et al. 2022; MOH 2021) as health workers reported being understaffed and overworked. Although there is a contrast in findings, MOH (2021) acknowledges that health worker distribution has tended to be positively skewed towards the cities and urban centres such as Accra and Kumasi. Accra is a city and yet, health workers here report being understaffed and overwhelmed. In the light of this acknowledgement by the MOH, where there are few health workers, the high workload results in increased stress, the development of chronic health conditions, exhaustion and an inability to provide quality care (Secci & Syed 2023). Camara et al. (2020) highlight that burnout not only affects the health of the caregiver but also potentially impacts negatively on patient health outcomes. What this translates to is the unpleasant experience that patients associate with hospital visits and increased exposure to ‘harmful’ care, which increases the overall cost of healthcare (World Bank Group 2018).

The study also found that the ability to pay, at the level of the individual, is one of the determinants of access to PCC. From the patients’ perspective, financial capacity influences the decision to make hospital visits (Umar, Fusheini & Ayanore 2021). Patients are unable to have continuity of care even where the NHIS is in place and works well because they encounter high out-of-pocket payments (Kipo-Sunyehzi 2021). In some cases, patients cannot even afford the cost of transportation to the health facilities (Kipo-Sunyehzi 2021). The additional out-of-pocket costs for healthcare despite the NHIS pose a significant challenge to the quest for UHC. Aikins et al. (2021) submit that the scheme would benefit from a comprehensive review of its benefits and coverage to serve the evolving long-term needs of the population. The fact that wealth is found to be a determinant to accessing healthcare in Ghana is cause for concern as this is divergent to the aim of UHC and the purpose for which the NHIS was set up (Umar et al. 2021). The findings support the call for a review of the benefits package made by Vellekoop, Odame and Ochalek (2022).

Inadequate financing at the organisational level is often evidenced by a lack of adequate medical equipment, consumables and poor maintenance of available equipment. This aligns with the conclusions drawn by Kportufe (2015) and Adem et al. (2023), who highlight the widespread issue of poor equipment and infrastructure maintenance.

Furthermore, the scarcity of essential service implements such as gloves and running water reflects poorly on leadership, posing potential sources of harm for patients and service providers.

Another important factor influencing PCC and PE is communication (Camara et al. 2020) point out that poor communication and oppressive language are experienced by many patients when interacting with healthcare systems in Africa. While communication skills can be learned, attitudes are difficult to change, and negative individual attitudes can lead to a breakdown of the provider-patient engagement (Kwame & Petrucka 2020). Participants in this study described how language barriers made it difficult to explain important information to patients, leading to negative implications for UHC.

Patients are increasingly becoming aware of their rights and this necessitates better quality health service. Patients in this study, described the negative attitudes of health workers, especially the rude way nurses spoke. This finding supports the conclusion drawn by Wu et al. (2021) who state that patient satisfaction is dependent on provider friendliness. This negative perception of health workers must be addressed systematically and deliberately to ensure quality care provision, especially because good communication is key to building trust, strengthening patient commitment and encouraging patient compliance.

Service structure, time spent waiting to see a doctor or access a service, referral system in place and ease of navigation of healthcare facilities are organisational-level factors that need consideration to foster PCC. Patients from this study revealed that they would wait for an average of 4 h to see doctors. The long waiting times were also described by another participant who shared that they now considered their hospital visit days as travel days, and therefore they come prepared to spend a lot of time at the hospital. The time patients spend trying to access healthcare can promote return visits and increase compliance or discourage them (Al-Harajin, Al-Subaie & Elzubair 2019). The findings of this study show that the time spent by patients waiting to obtain healthcare is important to the patient experience of quality care. The long waiting times are undesirable in fostering PCC although related studies (Ofei-Dodoo 2019; Sokoloff et al. 2020) show that these long waiting times do not affect patient satisfaction. Such long waiting times need to be addressed as timeliness is a feature of quality care and the right care must be on time to be of value.

### Limitations

While admitting that this study is timely in providing one aspect of the face of healthcare in Ghana, in seeking to identify and explore the factors that have influenced PCC and PE, it has not been able to cover all the factors that may be beneficial or inimical to the concepts. The study has been successful in presenting a balanced view from care providers and care recipients on what they consider important factors for healthcare quality.

## Conclusion

In setting out to identify factors that affect the access to PCC and PE from the viewpoints of healthcare providers and patients or clients, the study found that efforts to sustain progress towards attaining SDG 3 are present although plagued with challenges. These factors were grouped into three main categories within the context of Ghana:

- *Organisational Factors*: These include resource availability (e.g., equipment, consumables) and staffing levels, particularly highlighting health worker burnout. These challenges affect the capacity of healthcare facilities to provide timely and quality care.
- *Individual Factors*: This encompasses the attitudes, motivation and communication skills of healthcare workers. The quality of communication, which should convey respect, empathy and trust, plays a crucial role in shaping patient experiences and empowering them to make informed health decisions.
- *Environmental Factors*: Factors such as safety, security and cleanliness of facilities are essential to ensuring a conducive environment for both patients and healthcare workers. Poor facilities and geographical challenges, such as the state of the road networks, further impede access to healthcare.

The recommendations based on the factors identified, centre around improved resource efficiency, a commitment to deploying enhanced strategies for communication in health and a reduction in patient waiting times. Long waiting times negatively impact patient satisfaction and general productivity. Addressing this issue requires the development of efficient service delivery models to streamline patient flow and reduce the time patients spend waiting for care. By focussing on the factors outlined, the Ghanaian health system can make strides in enhancing PCC and empowering patients.
